# Genetic variation and function: revealing potential factors associated with microbial phenotypes

**DOI:** 10.52601/bpr.2021.200040

**Published:** 2021-04-30

**Authors:** Xiaolin Liu, Yue Ma, Jun Wang

**Affiliations:** 1 CAS Key Laboratory of Pathogenic Microbiology and Immunology, Institute of Microbiology, Chinese Academy of Sciences, Beijing 100101, China; 2 University of Chinese Academy of Sciences, Beijing 100049, China

**Keywords:** Genetic variation, Phenotype, Association analysis, Comparative analysis, Microbiome

## Abstract

Innovations in sequencing technology have generated voluminous microbial and host genomic data, making it possible to detect these genetic variations and analyze the function influenced by them. Recently, many studies have linked such genetic variations to phenotypes through association or comparative analysis, which have further advanced our understanding of multiple microbial functions. In this review, we summarized the application of association analysis in microbes like *Mycobacterium tuberculosis*, focusing on screening of microbial genetic variants potentially associated with phenotypes such as drug resistance, pathogenesis and novel drug targets *etc*.; reviewed the application of additional comparative genomic or transcriptomic methods to identify genetic factors associated with functions in microbes; expanded the scope of our study to focus on host genetic factors associated with certain microbes or microbiome and summarized the recent host genetic variations associated with microbial phenotypes, including susceptibility and load after infection of HIV, presence/absence of different taxa, and quantitative traits of microbiome, and lastly, discussed the challenges that may be encountered and the apparent or potential viable solutions. Gene-function analysis of microbe and microbiome is still in its infancy, and in order to unleash its full potential, it is necessary to understand its history, current status, and the challenges hindering its development.

## INTRODUCTION

Host-associated microbes play an important role in shaping the living circumstance and status of host. On one hand, microbes can beneficially help host digesting, breaking down food (Tasse *et al*. 2010), on the other hand, microbes can be pathogenic and lead to diseases even fatality in hosts. Diverse microbes make up microbiome whose composition and function are critical to host health and disease. Gut microbiome, for example, is increasingly being considered to influencing host health status, and the functional alterations of which can influence host in many ways, including an expanding list of diseases (Franzosa *et al*. 2014; Halfvarson *et al*. 2017). The identification of potential genetic variations responsible for functions of microbes or composition and functions of microbiome has therefore been concerned continuously.

Recently, the rapidly developing sequencing technologies have produced voluminous sequencing data that allows for association or comparative analysis to detect genetic variations and associate them with phenotypic diversifications. These analysis approaches, especially association analysis, have been being successfully applied in human (Andrews *et al*. 2020; Visscher *et al*. 2017), mice (Gonzales *et al*. 2018) and certain plants (Voichek and Weigel 2020; Zhang *et al*. 2019), which have made significant progress in understanding complex traits and biological mechanisms by identifying associated genetic factors of these organisms. Similarly, these methods have been used for detecting gene-function associations of single microbe, host-microbe and host-microbiome (Falush 2016; Falush and Bowden 2006; Power *et al*. 2017).

The success of genome-wide association analysis (GWAS) and quantitative trait loci (QTL) in human and other organisms made it gradually applied to microbial genomes, which has advanced comprehensions of microbial gene-function associations (Chapman and Hill 2012; Chen and Shapiro 2015; Falush 2016; Falush and Bowden 2006; Kurilshikov *et al*. 2017; Wang *et al*. 2018a). GWAS is frequently used to determine the genetic factors of drug resistance, virulence, host specificity and load of clinically relevant single microbes such as *Mycobacterium tuberculosis* (Table 1), HIV (Power *et al*. 2016; Power *et al*. 2017) *etc*., which can directly influence the severity and treatment of corresponding diseases of tuberculosis, AIDS. Microbiome has been concerned as well and microbiome GWAS (mGWAS) has been used to reveal host genetic influence on microbiome phenotypes, including the presence/absence (P/A) pattern and relative abundance of certain taxa and quantitative traits of microbiome. Furthermore, there is another GWAS named metagenome-wide association study (MGWAS) that aims to delineate associations using all sequencing data rather than the portion with species annotation information compared to mGWAS. Meanwhile, a concept of metagenomic linkage group (MLG) was generated to enlarge a taxonomic description (Qin *et al*. 2012). Additionally, another way of gene-function analysis used a combination of comparative genomics and transposon insertion sequencing to identify antibiotic resistance genes (ARGs) and reveal the mechanism of ARGs transmission among bacteria (Pal *et al*. 2016). In addition, comparative analysis of transcriptomic data collected under different conditions can be used to test the association between specific genes and functions.

**Table 1:** Examples of GWAS application in bacterial pathogens

Diseases	Species	Sample size	phenotype	Sig. viriants	Reference
Tuberculosis	*M. tuberculosis*	161	DR	*ponA1*	Farhat *et al*. 2013
		1526	DR	13 loci	Farhat *et al*. 2019
		161	DR	10 SNPs	Zhang *et al*. 2013
		1000	DR	*rpoA, B, C; eis*	Casali *et al*. 2014
		123	DR	58 SNPs	Chen and Shapiro 2015
		3651	DR	23 SNPs	Walker *et al*. 2015
		284	DR	*ald*	Desjardins *et al*. 2016
		6465	DR	43 SNPs	Coll *et al*. 2018
		710	DR	2 SNPs	Hicks *et al*. 2019
		177	DR	25 SNPs	Kavvas *et al*. 2020
Pneumonia	*S. pneumoniae*	3701	DR	51 SNPs	Chewapreecha *et al*. 2014
		1680	DR	4,317 SNPs	Mobegi *et al*. 2017
	*K. pneumoniae*	328	Virulence & DR	*wzi*	Holt *et al*. 2015
Pyomyositis	*S. aureus*	90	Virulence	121 SNPs	Laabei *et al*. 2014
		75	DR	1 SNPs	Alam *et al*. 2014
		518	Morbidity	PVL encoding genes	Young *et al*. 2019
Gastroenteritis	*C. jejuni*	192	Host adaptation	7 genes	Sheppard *et al*. 2013
		102	Biofilm formation	46 genes	Pascoe *et al*. 2015
		600	Survival	20 genes	Yahara *et al*. 2017
		166	Diagnostic Markers	25 genes	Buchanan *et al*. 2017
Meningitis	*Pneumococcus*	4572	DR & IPD	*pbp1b*A641C	Li *et al*. 2019a
DR: Drug resistance; IPD: Invasive pneumococcal disease.					

Functional analysis of microbial genes is an exciting field that uses the mounting microbial sequence data to reveal the ways in which genetic variation of microbes, as well as hosts affects bacterial pathogen, and microbiome phenotypes. The results of these studies have the potential to dramatically improve the way we understand, manage, and treat infectious diseases, as well as to increase our understanding of microbe-host interactions (Gilbert *et al*. 2016). However, unlike traditional human-based gene-function analyses, the high complexity of genetic content of microbes and the fact that their main pattern of proliferation is cloning. This pattern leads to a high level of linkage disequilibrium (LD) (Earle *et al*. 2016), making gene-phenotype association mapping of single microbe technically challenging. Furthermore, the complex composition of microbiome also poses an obstacle to localizing determinants of certain phenotypes to genes or genetic regions. In addition to association analysis, more accurate comparative analysis relies on increasingly precise genomic mapping and sequence analysis results. Therefore, additional and more effective tools are required for the gene-function analysis of microbes.

Studies of microbial gene-function analysis have provided many opportunities to researchers in recent years. However, the fact that this field is still at its early developing stage, with many bottlenecks and pitfalls, and requires continuous attention. This review summarized the application of association and comparative analysis to microbes, including the detection of microbial genetic variations associated with virulence, drug resistance, and host specificity and the determination of the interaction between certain microbes or microbiome and host genetic variations. Moreover, we analyzed the challenges that may be encountered in the study of microbial genetic variation and function, as well as apparent or potentially viable solutions.

## MICROBIAL GENETIC VARIATIONS INFLUENCING THEIR OWN FUNCTIONS

Gene-function analysis is essential for understanding microbial functions and microbe-host interactions. There are many approaches for gene-function analysis, such as the simple knockout-reversion assay, however it can only characterize function of one gene at a time. Large-scale association and comparative analysis allow for "high-throughput" analysis of gene-function linkages, which are of great interest for rapid and preliminary studies to understand the effects of genetic variation on function or phenotype.

### Association analysis revealing links of gene and function of individual microbe

**Figure 1:**
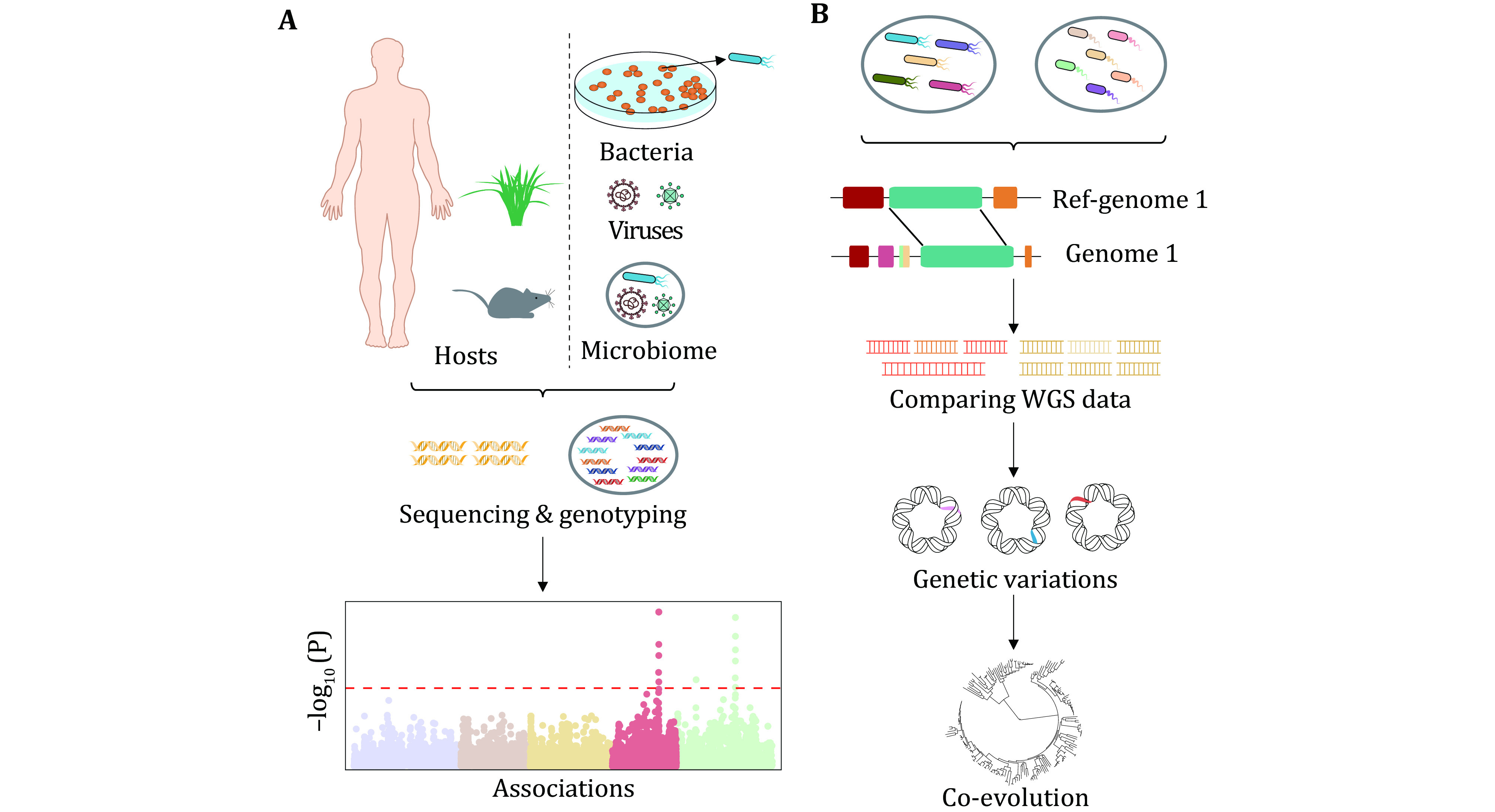
Overview of association and comparative analysis discussed in our study. **A** The outline of genome-wide association study (GWAS) of single microbes like pathogenic bacteria, uncovering the link between single microbial gene and function and the GWAS of host and single microbes like viruses or microbiome revealing the host-microbes gene-function relationship. **B** The outline of comparative analysis in single microbes such as some bacteria and yeast

Association analysis aims to link phenotypes, such as various clinical traits, and complex sets of features including taxa to genetic variations (Gilbert *et al*. 2016). For certain microbes, especially the clinically relevant pathogens, GWAS is predominantly used to detect their own genetic variants associated with traits like drug resistance, using the whole genome sequencing (WGS) data of microbial isolates (Fig. 1A), and the current development has indeed demonstrated its potential (Falush 2016).

There were numerous studies that have produced voluminous important insights into epidemiology, particularly for diseases such as tuberculosis (TB) caused by *Mycobacterium tuberculosis* (MTB). Tuberculosis is a severe disease caused by MTB and become difficult to treat due to its drug resistance potentially caused by genetic variations. therefore, identification of genetic determinants of drug resistance is very urgent for human disease and health. The conventional method for the detection of MTB drug resistance-associated genetic variants is the DNA banding assay represented by Genotype MTBDR, which can detect MTB resistance (*e.g*., rifampicin (RMP) and isoniazid (INH)) by DNA probe technology). Over a decade, this method has been tested, improved and optimized several times (Brossier *et al*. 2010; Hillemann *et al*. 2005; Jian *et al*. 2018; Lacoma *et al*. 2008). Although this method was highly accurate and performed well for the detection of known associations, it had little ability to detect new drug resistance-associated variants. Therefore, novel approaches were required to solve these conundrums, and then the potential of high throughput based GWAS effectively detecting associations was presented (Falush and Bowden 2006), and proofed by subsequent numerous studies.

**Figure 2:**
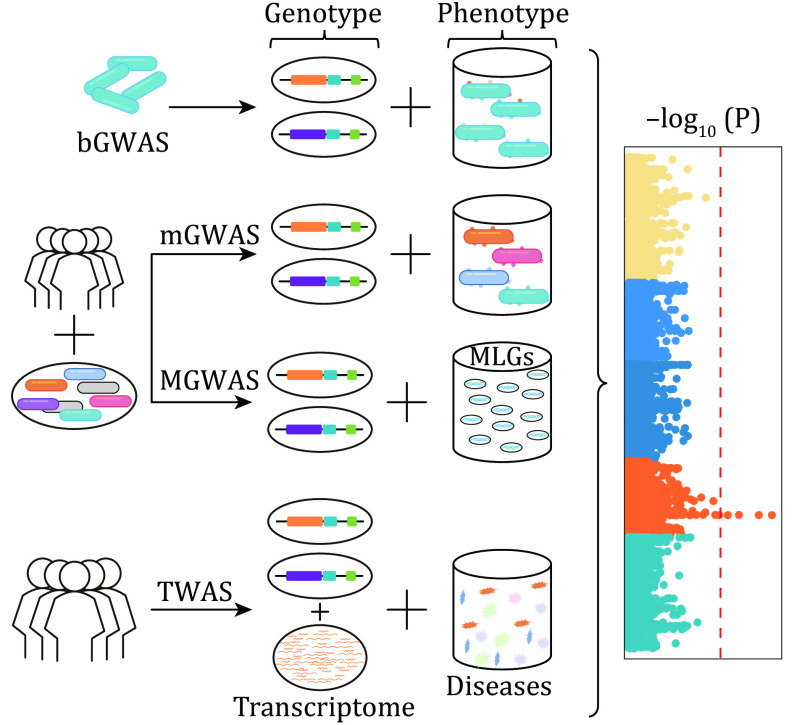
Overview of various association analyses. bGWAS, mGWAS, MGWAS, TWAS represent bacteria genome-wide association study, microbiome genome-wide association study, metagenome-wide association study, transcriptome-wide association study, respectively

Chan *et al*. reported a rapid genome-wide sequencing technique, which can significantly shorten the cycling time for genetic variant and associations detection of MTB isolates (Chan *et al*. 2013). Later, Farhat *et al*. collected 123 sequenced *M. tuberculosis* isolates genomes to seek for genetic loci associated with drug resistance and found *ponA1* that might associated with rifampicin resistance (Farhat *et al*. 2013). Thereafter, to continuously discover novel microbial genomic marker of drug resistance, they estimated the heritability of 1526 MTB isolates for resistance phenotypes to 11 antituberculosis drugs, eventually reporting 13 resistance-associated loci (Farhat *et al*. 2019). Conventional GWAS has identified many significant associations (Table 1). However, the high complexity of microbial genetic context has resulted in ample false positives in these results, and many studies have made progress on solving the puzzle. Zhang *et al*. sequenced and analyzed 161 MTB isolates collected in China, identified several genes and genetic regions associated with drug resistance, and constructed genome-wide phylogenetic tree to correct for possible effects of population structure (Zhang *et al*. 2013). Chen *et al*. and Walker *et al*. also combined GWAS with phyC (a phylogenetic tree construction tool) to detect drug resistance associations for 123 (Chen and Shapiro 2015) and 3651 (Walker *et al*. 2015) MTB isolates, and revealed 58 and 23 associations being detected, respectively. To gain a more comprehensive insight of the genetic basis of MTB resistance to antimicrobials in different regions, Desjardins *et al*. integrated WGS and phenotypic data on drug resistance from two large studies by Zhang *et al*. and Cohen *et al*. involving MTBs isolated from China and South Africa. They ultimately detected and experimentally demonstrated that the L-alanine dehydrogenase gene *ald* is a genetic factor for induction of novel drug resistance (Desjardins *et al*. 2016). Two other bGWAS (bacterial GWAS, Fig. 2) (Roe *et al*. 2020) on resistance of MTB to the second-line prodrug ethionamide (ETH) were also very effective in identifying certain genetic loci (Coll *et al*. 2018; Hicks *et al*. 2019). Kavvas *et al*. developed a genome-scale models (GEM)-based machine learning architecture to generate the datasets used in bGWAS and therefore increased the accuracy, ultimately identifying 25 significant associations (Kavvas *et al*. 2020). The identification of genetic factors of TB drug resistance is very urgent for epidemiology and therefore these findings provide very important insights for TB prevention and treatment.

In addition to bGWAS for MTB, numerous important bacteria have also been investigated. Pneumonia caused by *Streptococcus pneumoniae* is another common bacterial pathogenic infection and the widespread use of antimicrobial drugs has enabled it to acquire the resistance to many antimicrobial drugs, mainly beta-lactam antibiotics. Chewapreecha *et al*. performed bGWAS on 3701 *S. pneumoniae* isolates and found 51 genetic variants that may explain the beta-lactam antibiotics (Chewapreecha *et al*. 2014). Thereafter, Mobegi *et al*. conducted a bGWAS of 1680 *S. pneumoniae* isolates and identified possible genetic variation hotspots and demonstrated that these variation hotspots were associated with antibiotic resistance phenotypes (Mobegi *et al*. 2017). Several studies have also explored the potential genetic determinants of the phenotype of *S. pneumoniae* infection. For the carriage time, Lees *et al*. modeled longitudinal data on 598 unvaccinated children over a two-year period, and combined the data with WGS data to quantify and map the genetic factors of pneumococcal carriage time, showing that the *S. pneumoniae* genetic variation can explain most of the difference in carriage time (Lees *et al*. 2017). The study of Li *et al*. showed that *S. pneumoniae* *pbp1b* gene variation increases the chance of meningitis in infected individuals, *i.e*. pathogenicity (Li *et al*. 2019). Meanwhile, Lees *et al*. also found that the infective potential of *S. pneumoniae* is mostly explained by its own genetic variations, while its infection severity may be influenced by the genetics of its host (Lees *et al*. 2019). Another concerned bacterial infection is pyomyositis, which is a blood or muscle infection caused by *Staphylococcus aureus*, an opportunistic pathogen. However, the pathogenesis remains elusive until recently some studies have yielded many meaningful results. Some genetic loci affecting virulence (Laabei *et al*. 2014) and significant associations between vancomycin with Panton-Valentine leucocidin (PVL) locus (Alam *et al*. 2014; Young *et al*. 2019) were found. Furthermore, the comprehensions of link between PVL and septic myositis could hopefully reduce the occurrence of this disease by blocking PVL gene expression, which is of great clinical importance. One GWAS of invasive meningococcal isolates detected a gene (*pbp1bA641C*) is associated with the development of meningitis and drug resistance in *Pneumococcus meningitis* (Li *et al*. 2019a) (Table 1). Collectively, gene-function analysis of bacterial pathogens provides very important insights into the development and treatment of bacterial infectious diseases.

### Comparative analysis discovering links of gene and function in individual microbe

Comparative analysis of microbial genomic data usually contains comparing genomes of two or more subgroups to seek functional genes, evolutionary relationship and core gene clusters (Fig. 1B), and has produced many instructive insights in revealing the link between microbial phenotypes and genetic variations (Loeschcke 2013). Ormerod *et al*., for example, isolated 30 genomes of *Bacteroidales* S24-7 population from four different hosts (*Homo sapiens*, *Mus musculus*, *Phascolarctos cinereus* and *Cavia porcellus*) and then determined the evolutionary spectacle of S24-7 using comparative genomic analysis (Ormerod *et al*. 2016), demonstrating that comparative analysis can provide the first genetic insights into some uncultured gut-inhabiting bacteria. Reliy *et al*. compared genomes of 29 yeasts with promising application prospect and identified a genetic variant altering expression of *CUG-Ala*, a gene that coverts the standard leucine into alanine, which would severely affect the metabolic properties of *Pachysolen tannophilus* (Riley *et al*. 2016). In addition, comparative analysis can be utilized to investigate pathogenesis of disease occurrence with microbes, such as the contribution of molecular alterations in adherent-invasive *Escherichia coli* (AIEC) to Crohn's disease (O'Brien *et al*. 2017). The molecular mechanisms of regulation, such as regulation of SOS transcriptional response to DNA damage in (Sánchez-Osuna *et al*. 2017), and identification of microbial core genomes can be conducted using comparative analysis as well (Zhong *et al*. 2017).

Comparative analysis has also provided an important impetus to understand and manage the drug resistance in pathogenic microbes. ARGs determine the type and degree of antibiotics resistance of pathogenic microbes, and transposons are generally carriers of ARGs and are of great importance for the realization of horizontal gene transfer (HGT), the main potential factor accounting for the propagation of ARGs (Babakhani and Oloomi 2018; Berglund *et al*. 2017; van Hoek *et al*. 2011). Moreover, transposons are mobile DNA sequences moving around the genome by transcription or transposases (Wicker *et al*. 2007), causing genetic variations that may changes gene expression and shifts in a range of phenotypes (*e.g*., drug resistance, virulence). Therefore, transposons have been used as an important tool in gene-function analysis by generating libraries of functionally diverse mutants. Sequencing techniques then have been combined with these libraries to establish high-throughput transposon sequencing to identify genes involved in some biological processes (Barquist *et al*. 2013; Chao *et al*. 2016). For example, Eckert *et al*. established and sequenced a library of enterohemorrhagic *E. coli* (EHEC) transposon mutants and found 54 variants hitting 21 genes associated with gut microbiome formation of early life stage (Eckert *et al*. 2011). Recently, transposons were also combined with several advanced technologies, such as cell sorting and microfluidics that allows the encapsulation of individual transposon mutants into media-containing droplets for independent growth to associate complex unicellular traits with genetic variants (Thibault *et al*. 2019) and nanopore sequencing that can generate long reads with capability of covering entire transposons, allowing more accurate detection of gene variants and improved accuracy of gene-function analysis (Moss *et al*. 2020).

The transposon-based sequencing has been used in various cohorts such as infants (Gibson *et al*. 2016; Yassour *et al*. 2016), obese children (Wu *et al*. 2016), and Latin American low-income community cohorts (Pehrsson *et al*. 2016) to detect and manage ARG. In a study on *Mycobacterium* early on, conjugate transposons in bacteria were defined and recognized to be responsible for many ARGs transferring (Whittle *et al*. 2002). Recently, Cosials *et al*. presented the high-resolution structure of the Tn1549 Y transposase, which revealed the mechanism of transmission of resistance to vancomycin by Tn1549 conjugate transposons (Rubio-Cosials *et al*. 2018). The accumulation of ARG information has contributed to several databases (Alcock *et al*. 2019; Jia *et al*. 2017; Kleinheinz *et al*. 2014; Liu and Pop 2009), search engines (Rowe *et al*. 2015), and prediction tools (Arango-Argoty *et al*. 2018; Arango-Argoty *et al*. 2019; Yang *et al*. 2016).

## HOST GENETIC VARIATIONS INFLUENCING MICROBIAL TRAITS

Besides gene-function relationship in certain microbes, many work have also been performed on the hosts that have differences in responses against infections by microbes, and more importantly have a symbiotic relationship with microbiome for the majority of time. A mounting number of studies that combined microbe or microbiome and host genetic variations have emerged with developed sequencing technology, indicating that certain host genetic factors can account for microbial phenotypes.

### Association between host genes and single-microbial functions

In order to delineate how host genetic variations can impact the phenotypes of some viruses and other pathogenic microbes, numerous association analyses have been applied to these microbes. Taking HIV as an example, the global spread of AIDS caused by HIV is still not effectively controlled, and the toll of infections and deaths continues to rise, so it is urgent to gain a comprehension of the genetic mechanisms associated with HIV infection in human as well to control it. The co-evolution of HIV and human has led to HIV variability and made the development of treatments and vaccines challenging. To investigate the impact of this co-evolution, host genetic studies using candidate genes and genome-wide strategies have examined a variety of phenotypes, such as HIV susceptibility and viral load after infection (Chapman and Hill 2012). Felly *et al*. have contributed greatly to find host genetic factors associated with HIV phenotypes, mainly including polymorphisms within some chemokine receptor genes and SNPs on human leukocyte antigen (HLA) (Fellay *et al*. 2007, 2010; McCarthy *et al*. 2009). The applicability of existing GWAS to viral genome remains elusive, and approaches need to be confirmed or optimized in order to gain a more comprehensive and accurate understanding of various viral phenotypes (Power *et al*. 2016). For example, a method using genetic information of human infected with HIV and the pathogen collected respectively by genotyping and sequencing identified certain SNPs hit human HLA locus associated with diversity of viral amino acids (Bartha *et al*. 2013).

Similarly, confronting the rapid outbreak of coronavirus disease (COVID-19), researchers hoped to seek potential genetic factors for the development of COVID-19 through GWAS (Murray *et al*. 2020). In a work published recently, Ellinghaus *et al*. conducted GWAS on two cohorts from Italy and Spain. They identified a *3p21.31* gene cluster spanning a possible genetic locus associated with respiratory failure in patients, with possible involvement of the ABO blood group system (Ellinghaus *et al*. 2020). Although the results of association analysis require further validation to provide direct guidance for the prevention and treatment of COVID-19 infections, they contributed to providing alternatives. For non-viral microbes, there were also some researches that detected the host genetic variations impacting the microbial functions. In a study investigating genetic factors associated with the potential, susceptibility, and severity of *Streptococcus pneumoniae* infection, Lees *et al*. utilized the pneumococcal and host genomes data of MeninGene cohort (van de Beek *et al*. 2016) for combinatorial analysis (that is, combining human GWASs and bGWASs) to clarify the role of genetic variation in pathogens and host. The results suggested that genetic variation in the pathogen may be associated with invasive potential, whereas genetic variation in the host is associated with severity and susceptibility to pneumococcal meningitis (Lees *et al*. 2019).

### Association between host genes and functions of microbiome

Since the abundance of certain microbial taxa were identified to be influenced by host genetics in the twinUK cohort study (Goodrich *et al*. 2014), the microbiome GWAS (mGWAS, Fig. 2) was gradually utilized to detected host genetic variations impacting microbial phenotypes. The major phenotypes in mGWAS are bacterial taxa, microbial α-diversity and β-diversity *etc*. Some studies have combined these traits into microbial traits (MTs). Not only composition, but also functional metabolic pathways can be associated as phenotypes with genetic variation in the host genome. For metagenomic data, in order to thoroughly unleash the potential of these sequences rather than the portion with species annotation information, a concept of metagenomic linkage group (MLG) was generated to enlarge a taxonomic description. Furthermore, these MLGs were recognized as phenotypes in their metagenome GWAS (MGWAS) (Qin *et al*. 2012), which can be recognized as a branch of mGWAS (Fig. 2).

In recent years, mGWAS of human genetic context has revealed more than 300 associations (Table 2), most of which were studied for traits of microbial taxa (Kurilshikov *et al*. 2017). Incipiently, associations between the relative abundance of bacterial taxa and IBD risk genes were tested in a cohort containing 474 individuals, resulting in the identification of a significant association between nucleotide-binding oligomerization domain-containing protein 2 (*NOD2*) gene and the relative abundance of *Enterobacteriaceae* and the identification of an additional 48 IBD-related SNPs (Knights *et al*. 2014). Blekhman *et al*. found 83 associations with microbial taxa from ten body sites in a cohort (*n* = 93), including a possible association between *LCT* locus polymorphisms and the relative abundance of *Bifidobacterium* (Blekhman *et al*. 2015). Goodrich *et al*. pioneered the use of beta-diversity as another complementary phenotype, reporting 28 loci associated with bacterial taxa and three loci associated with microbiome beta-diversity in twinUK cohort (*n* = 1,126 twin pairs), which also reappeared the association between *LCT* gene and *Bifidobacterium* (Goodrich *et al*. 2016). Wang *et al*. used the same approach to detect associations in a cohort composed of 1812 individuals obtained from Popgen and Focus cohort, and they uncovered 42 loci associated with β-diversity, including encoding vitamin D receptor (*VDR*) gene, and 40 associations with bacterial taxa (Wang *et al*. 2016).

**Table 2:** Examples of mGWAS reveal association between host genetic variants and microbial traits

Cohort size	Host type	Phenotypes	Sig. variants	Sequencing	Reference
474	Human	Taxa	*NOD2* & 48 SNPs	16S	Knights *et al*. 2014
93	Human	Taxa	83 SNPs	16s	Blekhman *et al*. 2015
184	Human	Taxa	8 SNPs	16S	Davenport *et al*. 2015
1126 twin pairs	Human	β-diversity & taxa	31 loci	16S	Goodrich *et al*. 2016
1514	Human	Taxa & pathways	74 loci	WGS	Bonder *et al*. 2016
1561	Human	taxa	58 loci	16S	Turpin *et al*. 2016
1812	Human	β-diversity & taxa	82 loci	16S	Wang *et al*. 2016
725 twin pairs	Human	MTs	2 loci	16S	Demmitt *et al*. 2017
298	Human	Taxa	–	16S & WGS	Kolde *et al*. 2018
1882	Human	Taxa	–	16S & WGS	Rothschild *et al*. 2018
3880	Human	MTs	2 SNPs	16S	Hughes *et al*. 2020
18473	Human	Taxa	LCT gene	16S & WGS	Kurilshikov *et al*. 2020
1464	Human	Taxa & pathways	12 mbQTL	WGS	Hu *et al*. 2021
110	Mouse	Taxa	7 loci	16S	Org *et al*. 2015
196	*A. thaliana*	Taxa	–	16S	Horton *et al*. 2014

In addition to interpretation of host genetic determinants of microbiome composition, Bonder *et al*. introduced functional metabolic pathways into association analysis, and their mGWAS revealed nine taxonomically associated loci, 33 loci associated with pathways and 32 microbial quantitative trait loci (mbQTL) associated with complex diseases, innate and adaptive immunity, or food preferences (Bonder *et al*. 2016). Thereafter, Demmitt *et al*. introduced the concept of microbial traits (MTs), which include microbiome taxonomic groups, OTUs, α-diversity index, and β-diversity index, and they found two loci associated with MTs in 752 twin pairs (Demmitt *et al*. 2017). Then, taxa presence/absence (P/A) pattern, taxa abundance and enterotype were added to MTs by Hughes *et al.* and two significantly associated loci were reported as well (Hughes *et al*. 2020). Limitations caused by mGWAS based on only 16S data or WGS data alone made researchers sight to perform a conjoint analysis of these two data (Rothschild *et al*. 2018). Unfortunately, the result failed to provide a significant association. Similarly, Kolde *et al.* investigated association between genetic principal components of hosts and microbiome compositional and functional traits and many associations were found and the known association between *LCT* gene and abundance of *Bifidobacterium longum* in feces was reappeared (Kolde *et al*. 2018). Previous association analyses on microbiome using 16S and WGS sequencing data have profiled the host genetic factors associated with microbial taxa and their functional repertoire (Table 2). Qin *et al*. developed a protocol called MGWAS using metagenomic data of the gut microbiome from 345 Chinese individuals and then they detected and validated ~60,000 biomarkers associated with type 2 diabetes and established the concept of MLG, enabling thoroughly taxonomic species-level analyses (Qin *et al*. 2012).

Small cohort scale can contribute to lack of good overlap across studies and many pseudo-associations (Wang *et al*. 2018a). Therefore, Wang *et al*. proposed the MiBioGen consortium program, which convenes individual study cohorts and performs meta-analysis of the combined large cohort and is dedicated to providing a complete picture of human gene-microbiome associations (Wang *et al*. 2018b). By 2020, the program had included 25 large population cohorts containing a total of 18,473 individuals, and the GWAS meta-analysis revealed a significant association between the *LCT* gene and bacterial taxa (Kurilshikov *et al*. 2020). Not only adding insights into human genetic variation influencing microbiome traits, mGWAS has been successfully applied to *Arabidopsis* (Horton *et al*. 2014) and mouse (Org *et al*. 2015) as well. The biggest bottleneck of mGWAS is false positives caused by cohort scale. However, sequencing data accumulating can solve this difficulty and a mounting number of accurate associations are expected to be validated or detected.

However, mGWAS is still in its infancy, associations detected show small overlaps across studies because of factors such as analytical tools and microbial traits which are affected by some environmental factors such as gender, body mass index (BMI), and dietary fiber (Dominianni *et al*. 2015). Furthermore, the associations between host genetic variations and microbial composition and function is important for revealing complex diseases associated microbiome, such as inflammatory bowel disease and obesity. Directly association studies with disease reveal strong relationship between microbiome and ischemic heart disease, type 2 diabetes, obesity and insulin resistance (Kamada *et al*. 2013; Sanna *et al*. 2019; Yang *et al*. 2018). Furthermore, microbiome data can covariate with various data. The analysis of microbiome and clinical indicators revealed significant associations, such as human lipid levels altering microbiome (Falony *et al*. 2016; Fu *et al*. 2015). Microbiome composition and function were also detected to be influenced by some drugs (such as proton pump inhibitors and metformin) (Gorbunova *et al*. 2014; Imhann *et al*. 2018; Xu *et al*. 2018; Yoshii *et al*. 2019), which could alter gene expression by changing biogeography or environment (Weersma *et al*. 2020).

## DISCUSSION

The insights into genetic variation and microbial phenotype or function are meaningful for understanding and managing human health and disease. In this review, we retrospect the use of association and comparative analysis to resolve gene-function relationships, including the effects of single microbial genetic variation on their own virulence, drug resistance, load, and host adaptation, as well as the effects of host genetic variation on the composition and function of microbiome.

For associations between single microbial genetic variation and function, association and comparative analysis were mentioned. For association analysis, bGWAS is based on the successful application of GWAS in human and other model organisms, which has grown rapidly and now has its own very comprehensive database, the NHGRI-EBI GWAS Catalog (https://www.ebi.ac.uk/gwas/) being continuously updated with statistics of published studies. The bGWAS has indeed made significant advances in understanding the genetic mechanisms underlying clinically relevant traits and has uncovered many risk variants associated with resistance to multiple antibiotics or antimicrobials, providing many novel insights into the treatment and vaccines of infectious diseases. Nevertheless, the results across studies lack of overlap due to differences in detection approaches and sample sizes *etc.* Meanwhile, many clinically relevant phenotypes evolved under strong positive selection, so a relatively small sample size could theoretically be sufficient to identify causal variants (Manolio *et al*. 2009). However, the small sample size could pose some obstacles for detection ability of association analysis as well. For comparative analysis, we mainly reviewed the comparative genomics analysis with transposon because other factors that can cause structure variation in the genetic content of microbes, such as gene insertion by phages, generally do not cause the emerging of drug resistance. Greatly reducing complexity of bacterial isolates or microbiome metagenomic data, longer reads now can be generated by third-generation sequencing technology while its inaccuracy be corrected partly combined with NGS short reads. Furthermore, certain important genes, such as ARG, are located on plasmids or transposons. Besides sequence information, spatial information is also valuable. Attempts can be made to add spatial information collected by technologies like CHIP-seq (Chromatin Immunoprecipitation sequencing), which may provide more comprehensive insights in drug resistance with regard to gene and genetic structure and even modification (Boolchandani *et al*. 2019).

For associations between host genetic variation and microbial function, the association analyses have also been applied successfully and produced many important insights. In addition to the problems mentioned in bGWAS part, the population structure of microbes or microbiome increase the occurrence of false associations (Saber and Shapiro 2020). Therefore, optimizations in sequencing techniques and analytical methods are also in urgent demand. For GWAS detecting links between gene and function of certain microbes, SEER (Lees *et al*. 2016), pyseer (Lees *et al*. 2018), phyC (Farhat *et al*. 2013), treeWAS (Collins and Didelot 2018), pan-GWAS (Brynildsrud *et al*. 2016), and the introduction of machine learning (Kavvas *et al*. 2020) could increase the accuracy of association detection.

Additionally, there are numerous emerging methods for resolving microbial functions, especially at the expressional level. The transcriptome-wide association study (TWAS) can be utilized to establish the relationship between gene expression and traits that are genetically regulated (Wainberg *et al.* 2019), sharing part principles of GWAS and including transcriptomic information additionally (Fig. 2). TWAS has currently acquired some important achievements in human, mainly in elucidating the pathogenesis of complex diseases such as Parkinson's, Schizophrenia, chronic kidney disease and cancer (Feng *et al*. 2020; Gandal *et al*. 2018; Gusev *et al*. 2018; Hellwege *et al*. 2019; Li *et al*. 2019b). It is believed that with the development of transcriptome sequencing, this technology can also be successfully applied to the field of microbiology to further elucidate the association of microbial functions with genes and gene expression. Comparative transcriptome analysis is also important to determine the mechanisms of disease and physiology and has been successfully applied to human and mice (Breschi *et al*. 2017), which increased our understanding of relationship between phenotype or function and RNA information. This approach allows us to derive associations between traits and differential gene expression or modifications. In addition, by adding a time dimension, the method can be used to determine the regulatory factors and regulatory networks of a process (Chang *et al*. 2019). There were numerous studies that compared transcriptomic data collected from different microbes for identifying traits-associated expressional discrepancies such as resistance in *Pseudomonas aeruginosa*. RNA sequencing was conducted to identify genetic determinants of drug resistance in 135 clinical isolates from different geographic regions and infection sites, resulting in the identification of adaptive variants associated with fluoroquinolone, aminoglycoside, and β-lactam antibiotic resistance (Khaledi *et al*. 2016). Schniederjans *et al*. then analyzed *Pseudomonas* *aeruginosa* isolates with aminoglycoside resistance by combining comparative transcriptomics analysis and mutational profiling, suggesting that the phenotypes may be associated with activating in AmgRS and PmrAB (Schniederjans *et al*. 2017). Moreover, comparative transcriptomic analysis was applied to fungi to investigate the metabolism regulation by fungal RNA, such as development of fruiting body in filamentous *Ascomycetes* and metabolite production *etc*. (Lütkenhaus *et al*. 2019; Zhang *et al*. 2020).

In addition to protein-coding RNAs, Non-coding RNAs (ncRNAs) have been proved to be key regulatory elements of a wide range of cellular processes as well (Moody *et al*. 2013). Early on, a study using a comparative RNA sequencing analysis of three divergent model *Streptomycetes* (*S. coelicolor*, *S. avermitilis* and *S. venezuelae*) suggested that a number of ncRNAs might have regulatory control over antibiotic production in these bacteria (Moody *et al*. 2013). Another study showed that certain ncRNAs could regulate biological processes of cell wall to acquire drug resistance in *E. coli* (Fröhlich *et al*. 2012). The perspective that ncRNAs could modulate bacterial drug resistance have been gradually accepted (Dersch *et al*. 2017). Recently, the potential contribution of ncRNAs to drug resistance has become increasingly apparent. In particular, some small RNAs (sRNAs) may have implication of antibiotic response and resistance in some bacterial pathogens, suggesting that they may serve as innovative drug targets (Felden and Cattoir 2018). Specifically, these sRNAs can regulate the expression of outer membrane protein F (ompF) by pairing with mRNAs to induce translation inhibition and mRNA degradation, thus reducing the permeability to some antibiotics (Parker and Gottesman 2016). In addition to sRNAs, there are many other ncRNAs whose roles played in microbes needs to be further elucidated.

Gene-function analysis of microbes played an important role of comprehending for delineating functions or phenotypes of single microbes or microbiome and the human-microbiome interactions. The results generated by association or comparative analysis have provided important and novel insights, especially for the prevention and control of infectious and immune-related diseases, and have provided new rationales for treatment of these diseases. Additionally, combining association and comparative analysis can potentially detect more accurate gene-function relationships (Price *et al*. 2018). Future works would need to address the challenges hindering its development to unleash its full potential.

## Conflict of interest

Xiaolin Liu, Yue Ma and Jun Wang declare that they have no conflict of interest.
